# Editorial: FIN Special Issue on PREVIEW

**DOI:** 10.3389/fnut.2021.811541

**Published:** 2021-12-22

**Authors:** Jennie C. Brand-Miller, Anne Raben

**Affiliations:** ^1^The University of Sydney, Darlington, WA, Australia; ^2^Department of Nutrition, Exercise and Sports, Faculty of Science, University of Copenhagen, Copenhagen, Denmark; ^3^Clinical Research, Copenhagen University Hospital - Steno Diabetes Center Copenhagen, Herlev, Denmark

**Keywords:** diabetes, diabetes mellitus, diet, weight maintenance, protein, glycemic index, carbohydrate

Despite evidence from clinical trials that type 2 diabetes (T2D) can be prevented through intensive lifestyle interventions resulting in weight loss, the reality is that weight regain and weight “creep” are common. Published in 2010, the DiOGenes study identified two dietary factors together (modestly higher protein and lower glycemic index, GI) that were associated with short-term prevention of weight regain after prior weight loss (1). On this basis, the PREVIEW (PREVention of diabetes through lifestyle intervention and population studies in Europe and around the World) study was designed to determine if the benefits extended to prevention of T2D prevention and longer-term weight loss maintenance (NCT01777893).

The randomized controlled trial component of PREVIEW was a 3-year multicenter study carried out in eight countries with a 2 × 2 factorial design comparing two diets and two levels of exercise intensity (2). In total 2,326 adults aged 25–70 years, body mass intake BMI ≥25 kg/m^2^ and prediabetes were enrolled. In the first phase, participants were required to lose ≥8% of body weight in 8 weeks using total meal replacements to be eligible for the second phase of weight loss maintenance. Successful adults then received a behavioral intervention including instructions to follow *either* a conventional healthy diet [15% of energy (E) from protein, 55E% from carbohydrates, GI ≥ 56, and 30E% from fat] *or* the intervention diet (25E% from protein, 45E% from carbohydrates, GI ≤ 50, and 30E% fat). Within each dietary arm, they were further randomized to either 150 min/week of moderate physical activity or 75 min/week of intense physical activity. The interventions were delivered by nutrition and exercise professionals in a group setting with frequent visits during the first year and fading frequency in years 2–3. Food records were collected at baseline and at 6, 12, 24, and 36 months.

The main findings of PREVIEW were published in 2020 (3). In brief, 8 in 10 participants reduced body weight by ≥8% in the 8-week weight loss phase. Average weight loss was 11% and higher than expected. At the 12-month timepoint, 74% (*n* = 1,381) of participants remained in the study, and 52% (*n* = 962) at 3 years. Among the completers, the incidence of diabetes was much lower than anticipated—only 3 in 100 participants (*n* = 62) developed diabetes whereas 13.5% was predicted. The incidence was the same in centers where the dropout rate was low vs. those with a high rate. With such a low number of cases, there were no significant differences between the two diets, two exercise groups, or any of the four combinations. Interestingly, significantly fewer participants in the high protein groups achieved normal glucose levels at the end of the study (*P* < 0.0001).

Maintaining the target intakes of protein and GI over 3 years proved difficult—indeed all four groups tended to consume higher protein diets compared to baseline. Nonetheless, at the 3-year timepoint, almost half of the participants maintained a clinically significant weight loss of ≥5%, and one in four achieved ≥10%. A secondary, pooled analysis of all four groups revealed that participants in the lowest vs. highest tertiles of GI and dietary glycemic load (lower GI and lower carbohydrate intake) gained less weight and had lower glycated hemoglobin level at the end of the study (4). The differences were significant with and without adjustment for multiple confounders including energy intake, macronutrients, and fiber. Higher intake of protein (reported and estimated from urinary nitrogen) was also associated with lower weight regain and glycated hemoglobin (5). Further findings from PREVIEW have been published, including studies on populations (6), physical activity (7), sleep (8), behavioral factors (9), and brain reactivity (10).

This special issue of *Frontiers in Nutrition* includes six more studies drawn from PREVIEW. Zhu et al. investigated appetite control, including hunger and satiety assessed by recall of feelings during the previous week. From 12-month timepoint onwards, sensations of hunger decreased more in the higher protein-lower GI arm than the conventional diet arm. Hunger and satiety ratings correlated with changes in body weight at most timepoints, despite no difference in weight regain between the two diets or two physical activity groups.

Buso et al. also studied appetite in the Sydney cohort of PREVIEW (*n* = 136). Fasting plasma concentrations of total ghrelin (a hunger hormone), total PYY (a satiety hormone), and appetite sensations were measured at baseline, 8 weeks, and 6, 12, 24, and 36 months. Ghrelin levels gradually increased and PYY concentrations decreased during weight loss maintenance, but again there were no detectable differences between the diets.

Also in Sydney, Meroni et al. hypothesized that higher protein-lower GI diets may be more nutritious with more micronutrients than the conventional diet. Both diets were associated with comparable reductions in energy and saturated fat, and increases in dietary fiber. However, intakes of zinc, selenium, niacin, vitamin B12, and dietary cholesterol were higher in the higher protein-lower GI arm.

Tremblay et al. evaluated the profile of participants who were unable to achieve a body weight loss of ≥8% in response to the 8 week low-energy diet. The mean daily energy deficit of unsuccessful responders was only half that of successful participants, and they appeared to be more susceptible to hunger, stress, and poor sleep cues.

Finally, Navas-Carretero et al. studied the role of two novel indices that reflect insulin resistance: the Triglyceride Glucose Index and the Hypertriglyceridemic-High Waist phenotype. Higher baseline TyG index (*p* < 0.001) was associated with greater reductions in body weight, fasting glucose, and TG, while a high TyG-waist phenotype predicted better TG responses. These two indices may allow for a more personalized approach to prevention of T2D in susceptible individuals.

Taken together, the findings of PREVIEW indicate that a combination of rapid weight loss using total meal replacements, healthy diets, and physical activity during weight loss maintenance is highly effective for reducing the risk of T2D. Diets with moderately higher protein content and lower GI may be particularly helpful for appetite control in people with pre-diabetes.

## Author Contributions

JB-M and AR contributed equally to the writing of the Editorial manuscript.

## Conflict of Interest

JB-M is the President of the Glycemic Index Foundation, a not-for-profit food endorsement program. She receives royalties on books about the glycemic index of foods and oversees a glycemic index testing service at the University of Sydney. AR has received honorariums from the International Sweeteners Association and Unilever.

## Publisher's Note

All claims expressed in this article are solely those of the authors and do not necessarily represent those of their affiliated organizations, or those of the publisher, the editors and the reviewers. Any product that may be evaluated in this article, or claim that may be made by its manufacturer, is not guaranteed or endorsed by the publisher.
